# Supplementary material for: Genetic Analysis and Fine Mapping of a New Rice Mutant, Leaf Tip Senescence 2
Source: Int J Mol Sci. 2024 Jun 27;25(13):7082. doi: 10.3390/ijms25137082 (PMC11241029; doi:10.3390/ijms25137082)
Supplement: Supplementary file 1 [file ijms-25-07082-s001.zip › Additional file S1.pdf]

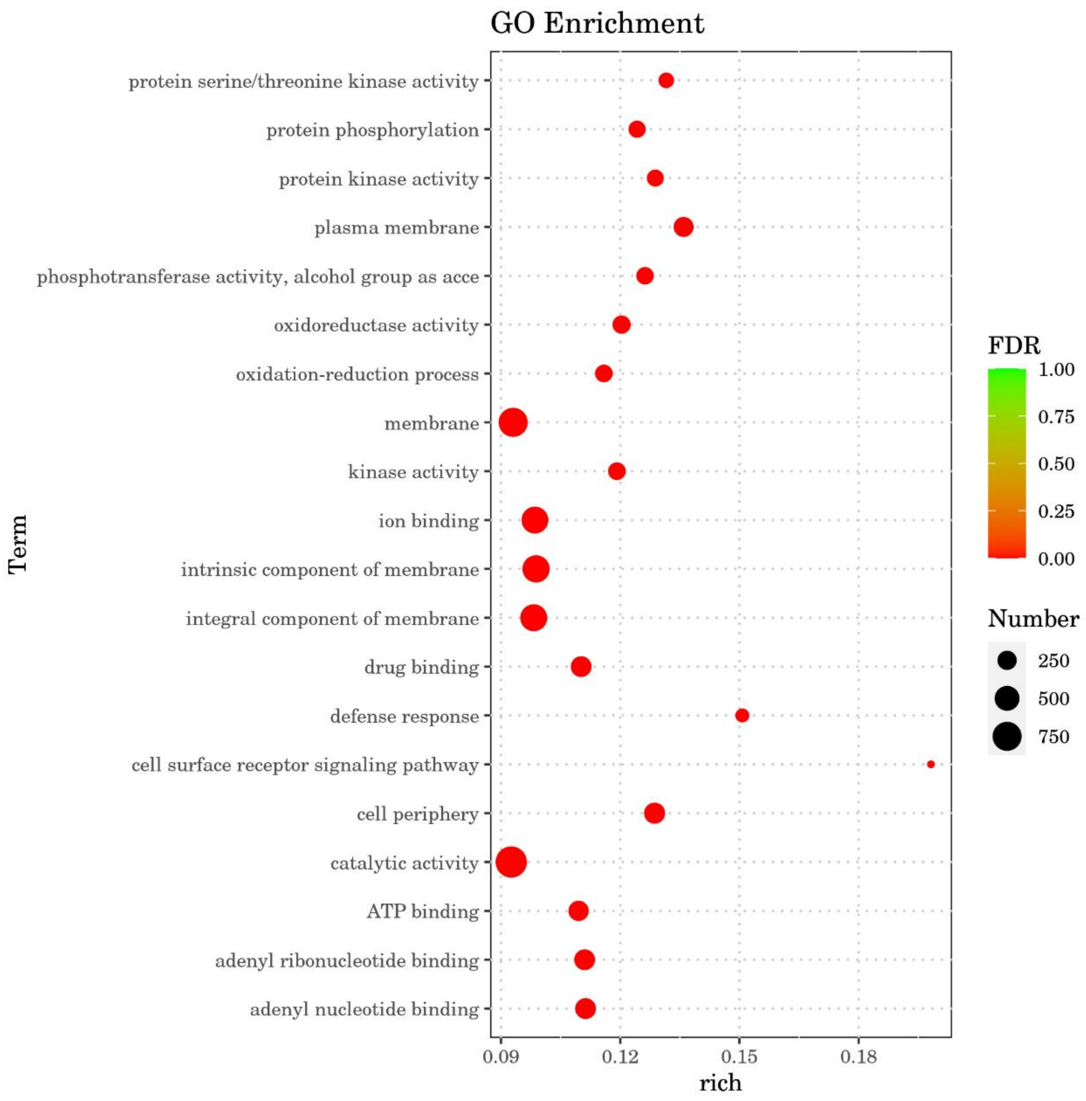

Fig. S1 Statistics of GO enrichment analysis of differential expression genes between wild-type(WT) and *lts2* plants. mRNA was purified from total RNA isolated from tillering-stage plants of WT ('changchungu') and *lts2*.

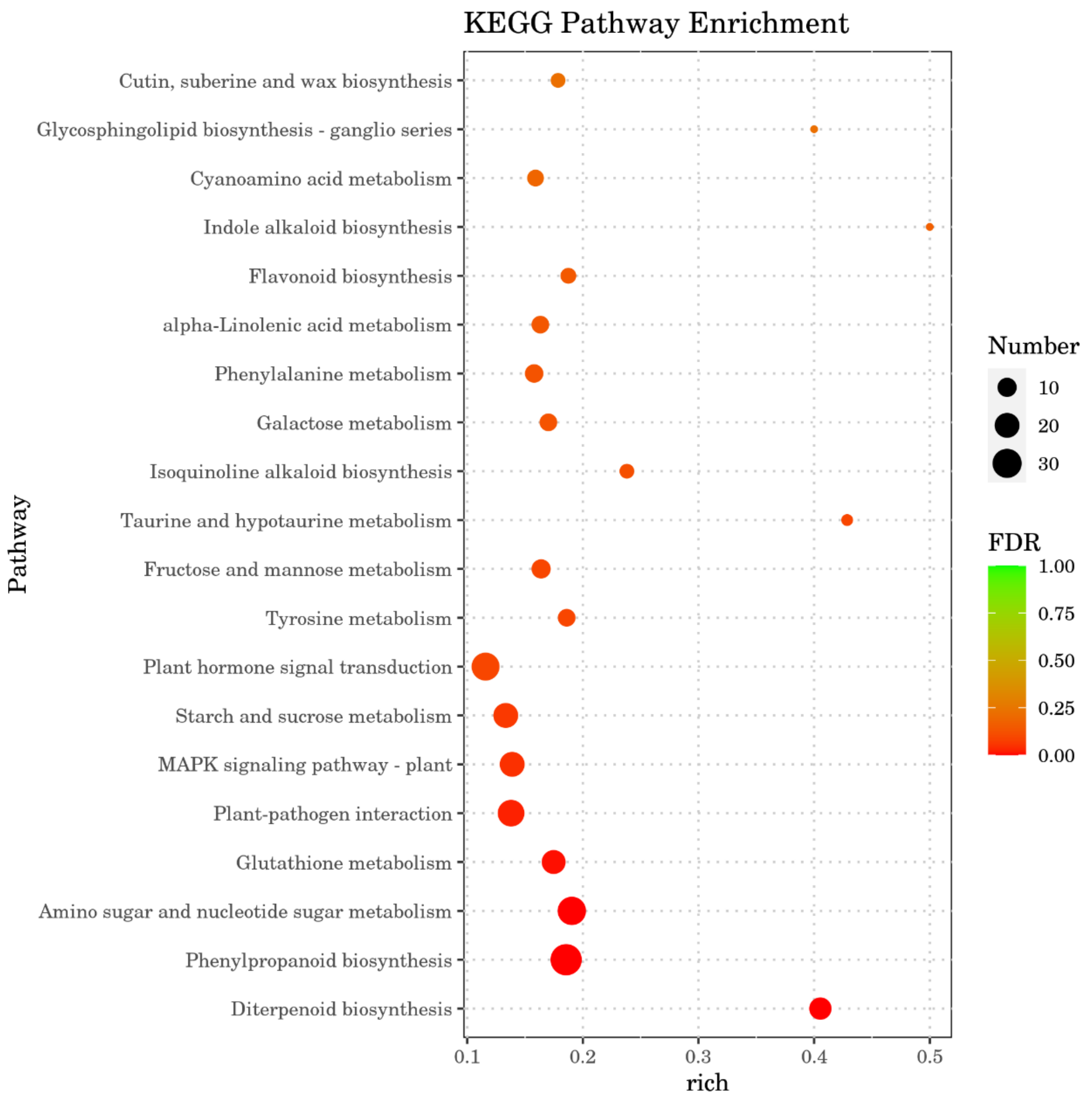

Fig. S2 Statistics of pathway enrichment analysis of differential expression genes between wild-type (WT) and *lts2* plants. mRNA was purified from total RNA isolated from tillering-stage plants of WT('changchungu') and *lts2*.

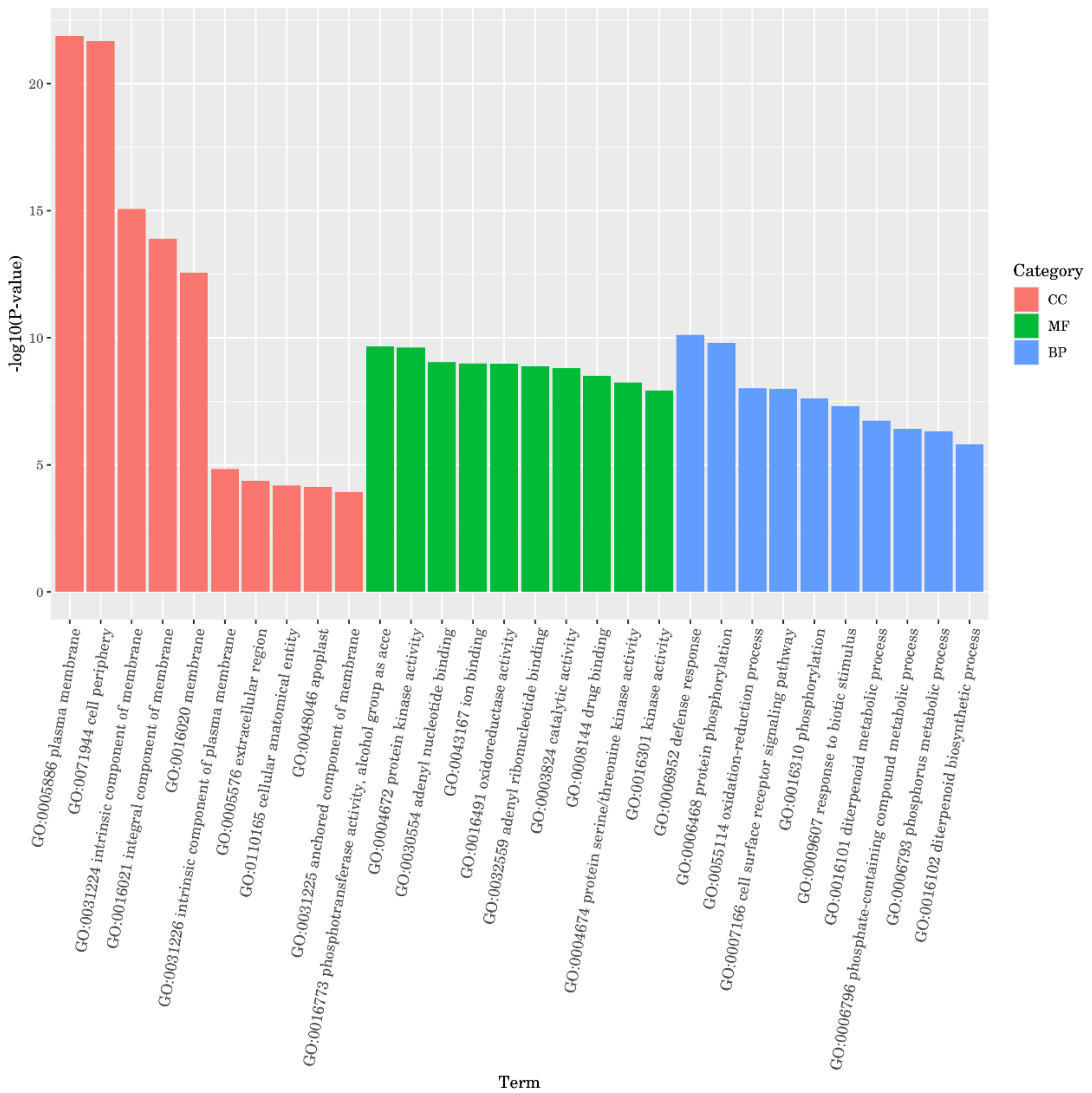

Fig. S3 The number of differential expression genes involved in different biological processes, cellular, components and molecular functions. mRNA was purified from total RNA isolated from tillering-stage plants of WT(‘changchungu’) and *lts2*.
